# Functional characterization of an arrestin gene on insecticide resistance of *Culex pipiens pallens*

**DOI:** 10.1186/1756-3305-5-134

**Published:** 2012-07-06

**Authors:** Yan Sun, Ping Zou, Xin-You Yu, Chen Chen, Jing Yu, Lin-Na Shi, Shan-Chao Hong, Dan Zhou, Xue-Lian Chang, Wei-Jie Wang, Bo Shen, Dong-Hui Zhang, Lei Ma, Chang-Liang Zhu

**Affiliations:** 1 Department of Pathogen Biology, Nanjing Medical University, 140 Hanzhong Road,, Nanjing, 210029, Jiang Su Province, People’s Republic of China

**Keywords:** Insecticide resistance, Arrestin, Gene cloning, Transfection, SiRNA, Cell viability

## Abstract

**Background:**

Continuous and excessive application of insecticides has resulted in the rapid development of insecticide resistance in several mosquito species, including *Culex pipiens pallens*. Previous studies in our laboratory found that arrestin gene expression was higher in the deltamethrin-resistant (DR) strain than in the deltamethrin-susceptible (DS) strain of *Cx. pipiens pallens.* Similarly, other studies reported that arrestin was highly expressed in permethrin-resistant *Cx. quinquefasciatus* and in dichlorodiphenyltrichloroethane (DDT)-resistant *Drosophila melanogaster.*

**Methods:**

Full-length cDNAs of an arrestin gene were cloned from *Cx. pipiens pallens* via polymerase chain reaction (PCR) and rapid amplification of cDNA end (RACE). The mRNA levels of the arrestin gene in the whole life cycle of DR and DS strains of *Cx. pipiens pallens* were investigated via quantitative real-time PCR. In addition, the relationship between arrestin and deltamethrin (DM) resistance were identified using genetic overexpression strategies and arrestin RNAi in mosquito cells. Cell viability was analyzed with cholecystokinin octapeptide after DM treatment. Moreover, the mRNA levels of cytochrome P450 6A1 (CYP6A1) and opsin in the transfected cells and controls were analyzed.

**Results:**

Complete arrestin gene sequence was cloned and expressed throughout the life cycle of *Cx. pipiens pallens*. Moreover, arrestin was significantly upregulated in the DR strain, compared with that in the DS strain at the egg, pupae, and adult stages. Arrestin overexpression comparably increased the mosquito cell viability, whereas arrestin knockdown by siRNA decreased mosquito cell viability with deltamethrin (DM) treatment. Meanwhile, the mRNA levels of CYP6A1 and opsin were upregulated in mosquito cells transfected with arrestin and downregulated in mosquito cells with arrestin knockdown.

**Conclusion:**

This study presented the first evidence that arrestin might be associated with insecticide resistance in *Cx. pipiens pallens*.

## Background

Mosquitoes are among the most important insect vectors that transmit numerous widespread and devastating insect-borne diseases, such as malaria [1], dengue fever [2], yellow fever [3], filariasis [4], Venezuelan equine encephalitis [5], West Nile fever [6], and chikungunya [7], thereby threatening public health. Vector-borne diseases account for about 17% of the estimated global burden of infectious diseases [8]. Therefore, considerable efforts have been taken to fight against these diseases, including drug development, vaccine research, and vector control [9]. Chemical control has been the main effective measure to reduce the population of these disease vectors since the 1950s [10]. Four classes of chemical insecticides are the mainstay of vector control programs, namely, organochlorines, organophosphates, carbamates, and pyrethroids. Pyrethroids account for approximately 25% of the world insecticide market and are used extensively because they kill insects rapidly and have low toxicity to mammals and birds [11]. Deltamethrin (DM), an important synthetic pyrethroid insecticide that kills insects by stimulating their nervous system (a similar mode of action to DDT), is widely used in bed net impregnation and indoor residual spray to help control the transmission of insect-borne diseases [12]. However, under natural selection, continuous and excessive application of DM and other synthetic pyrethroids have resulted in the rapid development of insecticide resistance in several species, including *Cx. pipiens pallens*. Insecticide resistance has become a major obstacle in the control of vector-borne diseases and a cause for a major public health concern [13].

Insecticide resistance is defined by the World Health Organization as "The development of an ability in a strain of an organism to tolerate doses of toxicants, which would prove lethal to a majority of individuals in a normal (susceptible) population of the same species." Elucidation of resistance mechanisms becomes crucial to guide the use of DM and the development of its substitutes, and should be considered one of the most challenging issues in modern applied entomology [14]. Resistance mechanisms in mosquitoes have been extensively studied and are known to be predominantly classified into two classes: metabolic resistance (degradation of the active ingredient by detoxification enzymes) and target site resistance (mutations in the target proteins). A number of genes associated with insecticide resistance, including cytochrome P450, esterases, GST, and sodium channel gene were identified [15,16]. Insecticide resistance is popularly known as a polygenetic phenomenon. Other genes contributing to the resistance are yet to be identified.

A large-scale transcriptional profiling based on suppression subtractive hybridization (SSH) combined with cDNA microarray has been completed in deltamethrin-susceptible (DS) and deltamethrin-resistant (DR) strains of *Cx. Pipiens pallens* in our previous experiments to investigate the DM resistance in *Cx. Pipiens pallens*. As a result, several genes associated with novel DM resistance have been isolated [17-19]. One of the highly expressed genes in the DR strain was opsin, which belongs to G protein-coupled receptors (GPCRs) [20]. Our further study on opsin involvement in insecticide resistance mechanisms accidentally revealed that arrestin gene expression was higher in the DR strain than in the DS strain of *Cx. pipiens pallens* (LC50 of DM, 0.50 mg/L vs. 0.02 mg/L)( Additional file 1: Figure S1). Similarly, arrestin was reported to be highly expressed in permethrin-resistant *Cx. quinquefasciatus*[21]. Arrestins are ubiquitously expressed proteins with basic biochemical mechanisms that include extracellular perturbation, specific receptors, coupling proteins such as G proteins, and effector enzymes or ion channels [22]. Recently, certain discoveries revealed new functions, such as chemotaxis [23,24] and metabolic regulation [25,26] carried out by the versatile arrestin. However, no studies related to insecticide resistance have been reported.

In this study, reverse transcription polymerase chain reaction (RT-PCR) and rapid amplification of cDNA end (RACE) were used to clone full length cDNAs of arrestin gene from *Cx. pipiens pallens*. The mRNA levels of arrestin gene in the DR and DS strains of *Cx. pipiens pallens* was then investigated via quantitative real-time PCR. The expression profile of the arrestin gene in the mosquito life cycle was also established. In addition, genetic overexpression and RNA interference strategies of arrestin in mosquito cells were used to identify the relationship between arrestin and DM resistance. Cell viability was analyzed after DM treatment. Cytochrome P450 6A1 (CYP6A1) is an important insecticide-resistant gene and opsin, one of the GPCRs, is related to DM resistance, thus, the mRNA levels of CYP6A1 and opsin in the transfected cells and controls were also analyzed. This study reports that arrestin gene is possibly involved in DM resistance.

## Methods

### Mosquitoes and cells

The DS strain of *Cx. pipiens pallens* was obtained from the Shanghai Institute of Entomology, Chinese Academy of Sciences and stored in our laboratory, without exposure to any insecticide. The DR strain was derived from the DS early fourth-instar larvae by selection with DM for more than 10 generations to reach a resistance 49-fold greater than that of the DS strain (LC50 of DM, 0.98 mg/L vs, 0.02 mg/L). Both the susceptible and resistant strains were reared in a 16 h light/8 h dark photoperiod at 25°C to 28°C. The C6/36 mosquito cell line was obtained from the China Center for Type Culture Collection (Wuhan, China). Cells were maintained in Dulbecco's modification of Eagle medium/high-glucose media supplemented with 10% (v/v) fetal bovine serum (Sijiqing, China) and 1% penicillin–streptomycin. The cells were grown in a humidified incubator with 5% CO_2_ at 28°C.

### RNA extraction and cDNA synthesis

Total RNA was extracted from every stage (egg, first, second, third, and fourth instar larvae, pupae male and female) of both DS and DR strains of *Cx. pipiens pallens* and mosquito cells using RNeasy the Mini Kit (QIAGEN, Hilden, Germany) according to the instructions of the manufacturer. The contaminating genomic DNA was removed via DNase I treatment. The quality of total RNA was determined by denaturing agarose gel electrophoresis, and the yield was estimated via spectrometry. The cDNAs were synthesized from 1 μg of total RNA using the PrimeScript™ RT reagent kit (TaKaRa, Shiga, Japan) according to the instructions of the manufacturer.

### Cloning and sequencing of arrestin cDNA

The open reading frame (ORF) of arrestin was cloned from the DR strain of *Cx. pipiens pallens* with specific primers 5'-ATGGTTTACAACTTCAAGGTCT-3' and 5'-CTAGTCAAAGTCAACCGACTGCT-3' designed according to the ORF of *Cx. quinquefasciatus* (GenBank ID:XM_001844435.1). 5'-RACE) and 3'-RACE were performed using the Clontech SMART™ RACE cDNA Amplification Kit (TaKaRa) according to the instructions of the manufacturer to clone the full-length cDNA of arrestin in *Cx. pipiens pallens*. The specific primers 5'-GCTGAGGGTAGACCTGCTCGGATGCC-3' (5'-RACE) and 5'-CTGGCATCCGAGCAGGTCTACC-3' (3'-RACE) were designed based on the aforementioned ORF (GenBank ID: HQ833831). The sequence of the 5'-RACE and 3'-RACE adaptor primers supplied by the kit was 5' -CTAATACGACTCACTATAGGGCAAGCAGTGGTATCAACGCAGAGT-3'. The fragment was separated via 1% agarose gel electrophoresis, purified using a quick gel extraction kit (QIAGEN), and was then ligated into the PCR2.1-T easy vector (Invitrogen, Carlsbad, CA, USA). The ligation mixture was transformed into Top10 *E. coli* cells, and the cells were streaked on lysogeny broth plates containing ampicillin (100 μg/ml). The positive colonies were selected and confirmed via PCR. Plasmid DNA was extracted using a plasmid mini kit (QIAGEN) and sequenced at Shanghai BGI. The sequencing results of 5' and 3' RACE were then assembled to generate a putative full-length arrestin cDNA. Degenerate primers 5'-GCAAGGAYTTYATGYTRAGCCC-3' and 5'-TTAGTCAAAGTCGACCGATTGCTGC-3' were designed by arrestin sequence alignment from several species to clone the partial ORF of arrestin from the mosquito cell line for the follow-up siRNA transfection. PCR was carried out using the Ex Taq Kit (TaKaRa), and the product was separated via 1% agarose gel electrophoresis and purified using the QIAquick Gel Extraction Kit (QIAGEN). Thereafter, the product was treated by thymine and adenine cloning. The positive colonies were confirmed via PCR and sequenced at Shanghai BGI.

### Sequence alignment and phylogenetic tree

The standard protein–protein BLAST sequence comparison and PSI-BLAST programs (BLASTP, http://blast.ncbi.nlm.nih.gov) were used to search for sequences in the Swiss-Prot databases with similarities to the translated arrestin protein. Deduced amino acid sequences alignment was analyzed with ClustalW2 program (http://www.ebi.ac.uk/Tools/clustalw2/index.html). The MEGA 5.0 program was used to analyze the phylogenetic tree via the neighbor-joining method [27]. The sequences included in our analysis for sequence alignment and phylogenetic tree were as follows: *Cx. pipiens pallens*, HQ833831.2; *Cx. quinquefasciatus*, XM_001844435.1; *Aedes aegypti*, XM_ 001663682.1; *Anopheles gambiae*, AY017417.1; *Drosophila simulans*, XM_002079727; *Drosophila yakuba*, XM_002090394.1; *Homo sapiens*, NM_004313.3; *Mus musculus*, NM_145429.4; and *Danio rerio*, NM_001159822.1. Arrestin isoelectric point and molecular mass were predicted using an online tool ExPASy (http://us.expasy.org/tools/pi_tool.html). Signal peptide and conserved domains were predicted with online tools SignalIP3.0 (http://www.cbs.dtu.dk/services/SignalP/) and SMART (http://smart.embl-heidelberg.de/). Cellular localization was analyzed with the online tool PSORT (http://psort.ims.u-tokyo.ac.jp/form.html).

### PCR and quantitative real-time PCR analysis

The PCR conditions for 5' and 3' RACE were as follows: initial denaturation at 95°C for 1 min, followed by 30 cycles at 95°C for 30 s, 68°C for 1 min, with a final 1 min extension at 68°C using KOD-Plus-Neo (TOYOBO). The PCR conditions for all other assays were as follows: initial denaturation at 94°C for 5 min, followed by 38 cycles at 94°C for 1 min, 56°C for 1 min, and 72°C 1 min, with a final 10 min extension at 72°C. Quantitative real-time PCR was performed on ABI PRISM 7300 (Applied Biosystems, CA, USA) with LightCycler FastStart DNA Master SYBR Green I (Rockford, IL, USA) according to the instructions of the manufacturer. The 20 μL PCR mixture contained 10 μL of 2 × SYBR Green PCR Master mix, 1 μL and 10 μM of forward and reverse primers, respectively, and 8 μL of diluted cDNA. Two pairs of primers were designed for this experiment: arrestin, forward 5' -GTCCAACAAGGAGCAGACCAAGC-3' and reverse 5'-ACTGGATCTTACGGATTCCCAGCGA-3', and *β*-actin, forward 5'-CGCTTCCTCGTCTACACTGG-3' and reverse 5'-GTGTTGGCGAACAGATCCTT-3'. All data were analyzed with a 7300 System SDS Software v1.2.1 (Applied Biosystems). The following conditions were employed: 50°C for 2 min, 95°C for 10 min, followed by 40 cycles at 95°C for 15 s and 60°C for 1 min. *β*-actin was used as internal control, and the threshold cycle (Ct) values were used to calculate the relative expression levels of each sample using the Relative Expression Software Tool 2008 [28]. Arrestin expression level at the egg stage of the DS strain was considered as background 1. All analyses for each assay were performed three times using independently purified RNA samples.

### Construction of eukaryotic expression plasmid

Arrestin ORF was amplified with a pair of specific primers: forward primer 5'-GGACTAGTGAGATGGAAATGGTTTACAACTTCAAGGTCT-3' and reverse primer 5'-GCCTCGAGAGCCTTCTCGTCGGAGGCG-3'. GAGATGGAA was added before ATG to form a Kozak sequence in the forward primer [29,30]. At the same time, the forward primer had an *Spe* I recognition site (ACTAGT) and the reverse primer had an *Xho* I recognition site (CTCGAG) to clone the ORF fragment into the pIB/V5-His expression vector. The PCR product and pIB/V5-His expression vector (Invitrogen, USA) were digested by *Spe* I and *Xho* I, and the two objective bands were then purified using the QIAquick Gel Extraction Kit (QIAGEN) and ligated with T4 DNA Ligase (NEB, USA). The ligation mixture was then transformed into TOP10 *E. coli* competent cells. The positive clones were identified via PCR with OpIE2 primers, forward 5'-CGCAACGATCTGGTAAACAC-3' and reverse 5'-GACAATACAAACTAAGATTTAGTCAG-3', supplied by the InsectSelect™ BSD System kit. The accuracy of the expression plasmid pIB/Arrestin was finally verified by sequencing.

### Transient transfection of arrestin

The pIB/Arrestin and pIB/Ctrl plasmids were transient-transfected into mosquito cells using FuGENE® HD Transfection Reagent (Roche, IN, USA) according to the instructions of the manufacturer. About 2 μg of plasmid DNA and 5 μL of FuGENE® HD Transfection Reagent were added separately to 0.5 ml of Opti-MEM when the cells reached about 75% confluence in a six-well plate. The two solutions were mixed gently and incubated at room temperature for 30 min. The entire mixture was then layered onto cells after being washed with Opti-MEM (Invitrogen, CA, USA). The cells were incubated with the mixture in a humidified incubator with 5% CO_2_ at 28°C, and the mixture was replaced with 2 ml of normal growth medium containing serum after a period of 6 h to 8 h. The cells were then incubated for an additional 48 h before harvest, and characterized via quantitative real-time PCR and Western blotting. The cell lines were collected for a subsequent assay after the cells were confirmed to have overexpressed the expected protein.

### Construction and transfection of siRNA

Corresponding to the arrestin cloned from mosquito cells, double-stranded siRNA molecules were designed and synthesized by the GenePharma Company (GenePharma, Shanghai, China). The sense sequences of the arrestin siRNA and scrambled siRNA control were as follows: 5'-CCTGCCUUCAGAAGGUUAUTT-3' and 5'-UUCUCCGAACGUGUCACGUTT-3', respectively. The mosquito C6/36 cells were transfected with siRNA using the FuGENE®HD Transfection Reagent according to the instructions of the manufacturer. siRNA (final concentration, 40 nM) and transfection reagent (6 μl) were mixed in 100 μL of Opti-MEM in a sterile and RNase-free tube for 15 min at room temperature, and were then added to the cells. The medium was replaced with fresh, complete media after incubation for 6 h at 28°C. Cells were collected for a subsequent assay after 24 h. The arrestin primers from mosquito cells were designed as follows: forward 5'-CAACAAGGAGCAGACCAAG-3' and reverse 5'-TGCAGAGTGATCGAGGATG-3' for quantitative real-time PCR. The β-actin primers were forward 5'- CCACCATGTACCCAGGAATC-3' and reverse 5'-CACCGATCCAGACGGAGTAT-3'.

### Western blot analysis

Proteins were extracted from pIB/Arrestin, pIB/Ctrl, siArrestin, and siCtrl-transfected cells with the use of RIPA lysis buffer (Biyotime, China) according to the instructions of the manufacturer. The concentrations were determined using the BCA Protein Assay Kit (Pierce, USA). Up to 40 μg of protein per lane was loaded in 10% sodium dodecyl sulfate polyacrylamide gel electrophoresis (SDS-PAGE) gel. The SDS-PAGE electrophoresis was run at 80 V for 30 min and 120 V for 60 min. The proteins were then transferred to a polyvinylidene fluoride membrane for 55 min at 300 mA using the Trans-Blot SD Cell and Systems (Bio-Rad, CA, USA). The fusion protein was detected using an anti-His monoclonal antibody (1:200; NovaGen, USA) and a peroxidase–conjugated goat anti-mouse secondary antibody (1:1,000, BD, USA). The siArrestin protein was detected using the anti-Arrestin monoclonal antibody (1:200; NovaGen, USA) and a peroxidase–conjugated goat anti-mouse secondary antibody (1:1,000, BD, USA), whereas the anti-tubulin monoclonal antibody (1:1,000, BD, USA) was used as an internal control. Detection was conducted using BeyoECL Plus (Pierce) according to the instructions of the manufacturer.

### Cell viability assay

Cell Counting Kit-8 (CCK-8, Dojindo, Japan) was used to determine cell viability (pIB/Arrestin transient transfected, pIB/Ctrl transient transfected, siArrestin transfected, and siCtrl transfected cells) under DM treatments. Cell suspension (100 μL) was distributed (5000 cells/well) in a 96-well plate and were pre-incubated in a humidified incubator with 5% CO_2_ at 28°C for 24 h. Cells were treated with 100 μL of DM at varying concentrations (final concentrations: 0, 12.5 μM, 39.6 μM, 125 μM, 396 μM, and 1250 μM). Approximately, 10 μL of CCK-8 solution was added to each well after 72 h. Subsequently, the plates were incubated for 5 h and absorbance was measured at 450 nm using a microplate reader. DM was dissolved in DMSO (Sigma, USA) and the final concentration of DMSO was 0.5% (v/v). The procedures were repeated three times using independent samples.

### Detection of CYP6A1 and opsin mRNA levels

Real-time quantitative PCR was performed to detect various levels of CYP6A1 (GenBank accession no. FJ423553) mRNA and opsin mRNA between pIB/Arrestin and pIB/Ctrl transfected cells as well as between siArrestin and siCtrl transfected cells. The sequences of forward and reverse primer for CYP6A1 and opsin were as follows: 5’-GGCCTCCAGCAGCATTCAT-3’ and 5’-TCACGATGCATGGACCAGAT-3, and 5’-CAGGCGGGTAATGGAAACA-3’ and 5’-ACATCATCAGGAAATCGGAGAA-3’, respectively. *β*-actin gene from mosquito cells was used as internal RNA control. The expression level of CYP6A1 or opsin in the control was considered as background 1, and the procedure was repeated three times using independent samples.

### Statistics

All results were means of three independent experiments. The quantitative real time PCR data were analyzed using the hypothesis test. Student's *t* test was used to analyze other data. A *p* value of <0.05 was considered significant.

## Results

### Cloning the full length cDNA of arrestin and Sequence analysis

Arrestin sequence was amplified by RT-PCR with 3’-RACE and 5’-RACE, as previously described. The full length of arrestin from *Cx. pipiens pallens* was 1,650 bps and was reported to GenBank (GenBank ID: HQ833831). The ORF of arrestin was 1,155 bps and encoded a 384 amino acid protein. Start codon ATG was found at 186–188 and the same frame stop codon TAG was at 1338–1340 with tailing signal sequence “AATAAA” and a poly (A) presented at the 3’-untranslated region. Meanwhile, the same-frame stop codon TAA was found at 162–164 upstream of start codon ATG, indicating that the sequence is the full length of arrestin mRNA ( Additional file 2: Figure S2). The isoelectric point of translated protein was 8.04 and the molecular mass was 43090.23, as predicted by ExPASy. Amino acid sequence alignment using the ClustalW2 software showed the conservation of arrestin in different species ( Additional file 3: Figure S3) [31]. The phylogenetic relationships of arrestin between *Cx. pipiens pallens* and other species showed that arrestin from *Cx. pipiens pallens* has the highest homology with *Cx. quinquefasciatus* and *Anopheles gambiae* using the neighbor-joining method ( Additional file 4: Figure S4). SMART showed that there are two domains within this protein, namely, pfam: Arrestin_N domain from 17 to 174 and pfam: Arrestin_C domain from 193 to 351. No signal peptide was found, and the cellular localization analysis indicated that arrestin possibly existed in the cytoplasm.

### Expression of arrestin gene at various developmental stages of DR and DS *Cx. pipiens pallens*

**Figure 1:**
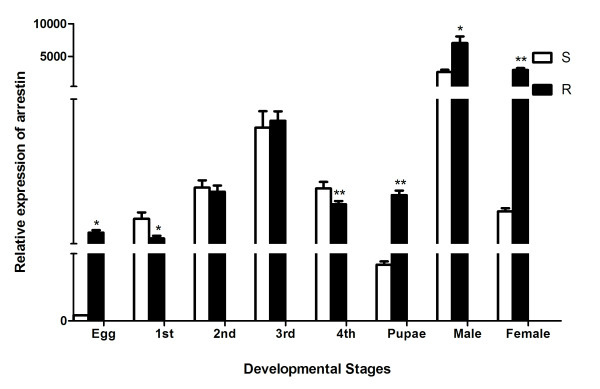
**Quantitative real-time PCR analysis of arrestin at different developmental stages of*Cx. pipiens pallens*.** The raw threshold cycle (Ct) values were normalized against standard *β*-actin to obtain normalized Ct values. The expression level of arrestin in susceptible strain eggs was considered as background 1. The data are means ± SD of three independent experiments. *p < 0.05,**p < 0.01.

Real-time quantitative RT-PCR was used to analyze the expression levels of arrestin in every stage (egg, 1st, 2nd, 3rd, 4th instar larvae, pupae male and female) of both DS and DR *Cx. pipiens pallens s* strains. A 242 bp cDNA fragment was selectively amplified using specific primers. The cycle number of arrestin at which the amplification reached its threshold was normalized against *β*-actin cycle number to determine the relative copy numbers between DR and DS strains. The maximum level of arrestin mRNA was detected in adult male mosquito, followed by adult female mosquito, the 3rd instar larvae, and the pupae. The transcriptional level of arrestin was up-regulated and exhibited 36-fold higher at the female stage in DR strain than in DS strain (Figure 1).

### Characterization of over-expression and silencing of arrestin in mosquito cells

**Figure 2:**
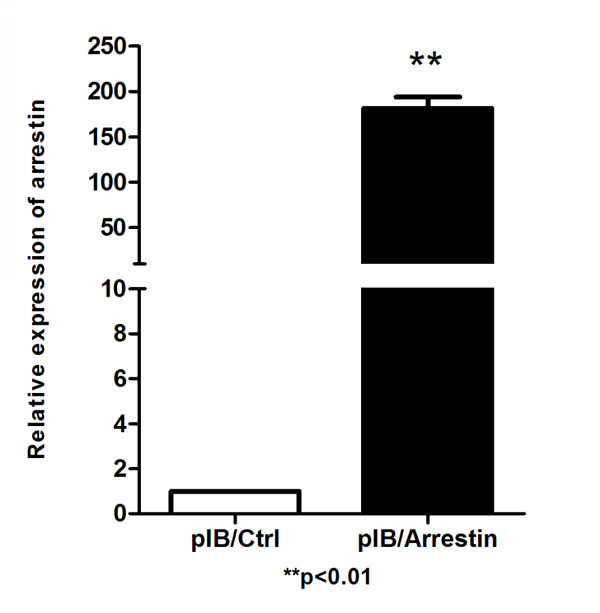
**Quantitative real-time PCR analysis of the overexpression efficiency of arrestin in cells.** Total RNAs (1.0 μg) from pIB/Arrestin cells and pIB/Ctrl cells were analyzed to detect arrestin expression. *β*-actin was used as internal control. n = 3; **p < 0.01. The experiment was repeated three times.

**Figure 3:**
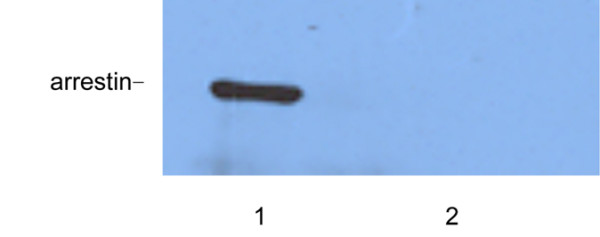
Western blot analysis of arrestin expression in cells transfected with pIB/Arrestin and pIB/Ctrl. 1: pIB/Arrestin; 2: pIB/Ctrl.

**Figure 4:**
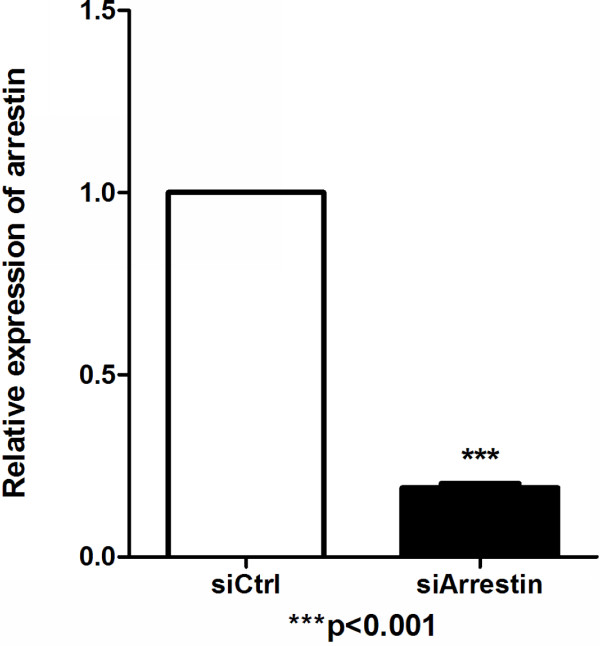
**Quantitative real-time PCR analysis of the knockdown efficiency of arrestin in cells.** Total RNAs (1.0 μg) from siCtrl transfected and siArrestin transfected cells were analyzed. *β*-actin was used as internal control. n = 3; ***p < 0.001. The experiment was repeated three times.

**Figure 5:**
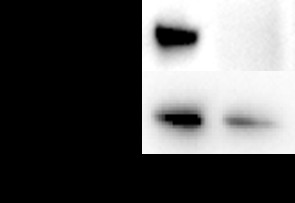
Western blot analysis of arrestin expression in cells transfected with siCtrl and siArrestin. 1: siCtrl; 2: siArrestin.

Quantitative real time PCR and Western blot analysis were conducted in the transient transfection experiment to confirm the transfection and expression of exogenous arrestin efficiency. Total RNA was isolated from arrestin transfected and control cells, where quantitative real time PCR was performed using arrestin specific primers. A pair of *β*-actin primers from mosquito cells was used to ensure the validity of the reaction system. Quantitative real-time PCR results showed that the expected fragment (242 bp) of arrestin was clearly over expressed in cells transfected with pIB/Arrestin vector of about 180.6-fold higher than that of control (Figure 2). Furthermore, a band of about 50 kDa was detected in pIB/Arrestin cell lysate through Western blot analysis using anti-his antibody (Figure 3). Altogether, these results indicated that exogenous arrestin had been expressed successfully in arrestin-transfected cells. Meanwhile, quantitative real time PCR was performed to examine the knockdown efficiency of arrestin-siRNA. Three siRNAs were designed by GenePharma Company and the most efficient one was chosen (data not shown). siRNA significantly down-regulated the expression level of arrestin (81% knock down) compared with the scramble siRNA control (Figure 4). Western blot analysis using anti-arrestin antibody suggested that arrestin was knocked down successfully (Figure 5).

### Viability assay of mosquito cells

**Figure 6:**
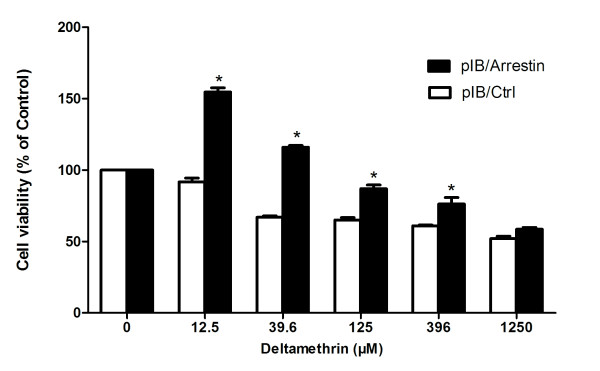
**Overexpression of arrestin enhances DM resistance in mosquito cells.** Transfected cells of pIB/Arrestin and pIB/Ctrl were treated with DM at indicated concentrations. Viable cells were measured by CCK-8 after 72 h of treatment. The percentage of viable cells is shown relative to the control (absorbance value of 0 μM). Results were expressed as mean ± standard deviation (SD) of triplicate wells from one out of three representative experiments. *p < 0.05 compared with pIB/Ctrl.

**Figure 7:**
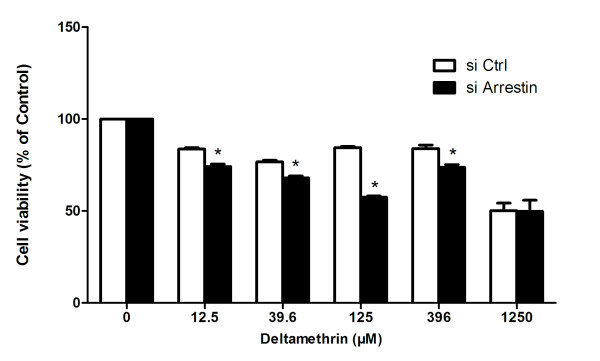
**siRNA mediated arrestin silencing reduces DM resistance in mosquito cells.** Transfected cells of siArrestin and siCtrl were treated with DM at indicated concentrations, and viable cells were measured by CCK-8 after 72 h of treatment. The percentage of viable cells is shown relative to the control (absorbance value of 0 mg/l). Results were expressed as mean ± standard deviation (SD) of triplicate wells from one representative experiment out of three. *p < 0.05 compared with siCtrl.

pIB/Arrestin and pIB/Ctrl transient transfected mosquito cells were used for cell viability assays to investigate the relationship between arrestin expression and DM resistance. The pIB/arrestin transfected cells were less susceptible to DM at 12.5 μM, 39.6 μM, 125 μM, and 396 μM concentrations compared with pIB/Ctrl groups (*p < 0.05) (Figure 6). Subsequently, arrestin-targeted siRNA in mosquito cells was further transfected as a reverse authentication, and cell viability analysis was then conducted. Cell viability in the siArrestin groups was lower than that of the siCtrl groups at the above four concentrations (*p < 0.05) (Figure 7), suggesting that arrestin possibly causes the mosquito cells to be more resistant to DM.

### CYP6A1 mRNA level and opsin mRNA level analysis

**Figure 8:**
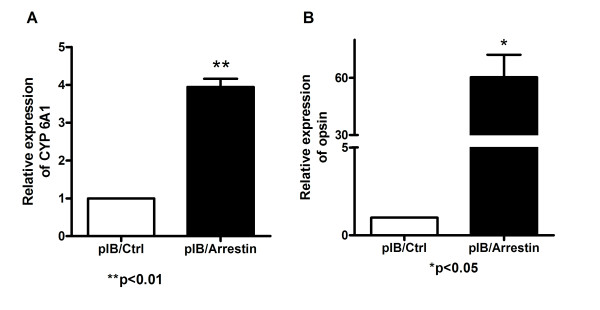
**Real-time quantitative PCR assay of CYP6A1 and opsin in pIB/Arrestin and pIB/Ctrl cells.** The relative expression of CYP6A1 or opsin in pIB/Ctrl cells was considered as background. Results were expressed as mean ± standard error. *β*-actin was used as internal control. n = 3; *p < 0.05, **p < 0.01. The experiment was repeated three times. A: CYP6A1 expression level; B: opsin expression level.

**Figure 9:**
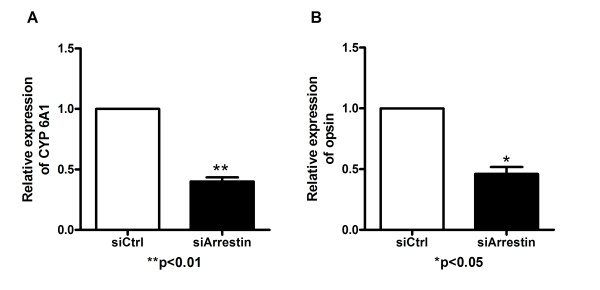
**Real-time quantitative PCR assay of CYP6A1 and opsin in siCtrl and siArrestin cells.** Results were expressed as mean ± standard error. The relative expression of CYP6A1 in siCtrl cells was considered as background level or 1. *β*-actin was used as internal control. n = 3; *p < 0.05, **p < 0.01. The experiment was repeated three times. A: CYP6A1 expression level; B: opsin expression level.

The mRNA level of CYP6A1 and opsin in pIB/Arrestin transfected cells was 3.94 and 60.69 -fold higher than that of the control, respectively (Figure 8), and both were 2.17 fold lower than that of control in siArrestin transfected cells compared with control (Figure 9). Therefore, the expression of arrestin has a positive correlation with that of CYP6A1 and opsin in mosquito cells.

## Discussion

The continuous and widespread use of insecticides results in the development of insecticide resistance, which is an ongoing challenge in the prevention of insect-borne diseases [32]. Identifying novel resistance genes and revealing the potential resistance mechanisms have become focal issues in the field of insect vector resistance. In this study, the full cDNA of arrestin from *Cx. pipiens pallens* was obtained at 1,650 bps. The putative amino acid sequence shares 99%, 92%, and 71% relevance with arrestin genes from *Cx. quinquefasciatusAnopheles gambiae*, and *Drosophila melanogaster,* respectively. Meanwhile, two domains have been observed within this protein, namely, pfam: Arrestin_N domain from 17 to 174 and pfam: Arrestin_C domain from 193 to 351. Despite the absence of similarity in any sequence, the two domains shared a very similar fold, known as the “arrestin fold” [33]. Arrestins are 48 kD to 55 kD proteins that have highly conserved structure (70% identity). Arrestins have a common constant amino acid sequence in the N-terminus, including the activate identification zone, phosphorylation recognition region, and the second hydrophobic interaction district, which enhances the affinity between arrestins and GPCRs [34]. All these data confirm that the gene obtained from *Cx. pipiens pallens* was arrestin.

The understanding of how resistance evolves at the molecular level is known predominantly to be involved in amplification, over-expression, and coding sequence variation of genes related to mechanisms of insecticide resistance [35,36]. Over expression of genes possibly accounts for the relative importance of conferring resistance [37]. Daborn reported that the up-regulation of CYP6Z1 gene led to DM resistance in *A. gambiae*[38] and the up-regulation of CYP6G1 gene resulted in DDT resistance in *D. melanogaster*[39]*.* CYP6A1 gene was over-expressed 12 and 10 fold in adults and larvae of diazinon-resistant *M. domestica*[40]. Insecticide resistance is a complicated genetic phenomenon and is involved in the multi-mechanism or interaction of several genes [41,42]. In this study, arrestin gene was up-regulated in the DR strain at the egg, pupae, male, and female stages. Over-expression of arrestin in mosquito cells increased their resistance to DM. Meanwhile, arrestin knockdown by siArrestin transfection decreased DM resistance. The result that arrestin can affect cell sensitivity to DM indicates that arrestin probably is a DM resistance related gene. Arrestin can regulate the expression levels of some genes in at least two ways: 1) Arrestin transfers from the cytoplasm to the nucleus and provides a scaffold for the transcription factor in the promoter region of target genes, which is called direct regulation; 2) Arrestin combines transcription regulation factors to change their activity and sub-cellular distribution, which is called indirect regulation [43]. Therefore, arrestin possibly affects some insecticide-resistant genes by regulating gene transcription.

The transcriptional levels of CYP6A1 in the corresponding cells were examined to further investigate the mechanism of arrestin in DM resistance. CYP6A1 is one of the cytochrome P450 family members, which plays an important role in insecticide resistance and was first isolated from diazinon-resistant *M. domestica*[44]. Some researchers found that CYP6A1 was expressed significantly higher in pyrethroid-resistant strains than in susceptible strains from other insects [40,41]. In our previous study on the relationship of ribosomal protein L22 and DM resistance, CYP6A1 was also found to be up-regulated in mosquito cells resistant to DM [45]. In this study, the expression levels of CYP6A1 were up-regulated and down-regulated when arrestin was over-expressed and knocked down. These changes were positively correlated with cell viability variations. Therefore, arrestin is possibly related to DM resistance by affecting the transcriptional levels of CYP6A1.

Some reports revealed that arrestins contribute to morphine tolerance through desensitization and internalization of opioid receptor (one member of GPCRs) [46-49]. Opsin, another member of GPCRs, was found to be over expressed in DDT-resistant *D. melanogaster* and several pyrethroid-resistant mosquitoes [21,50]. Opsin is a DM resistance related gene identified in our previous study [20]. In Classic GPCR signaling pathway, opsin and arrestin change their conformations, bind or separate with each other, and affect the downstream signaling [51,52]. In this study, the expression levels of opsin were up-regulated and down-regulated when arrestin was over expressed and knocked down in mosquito cells. These changes were positively correlated with cell viability variations. Therefore, arrestin and opsin are essential in DM resistance through the GPCR pathway.

In summary, our results provided evidence, for the first time, that arrestin might be a resistance relative gene. In addition, whether arrestin participates in resistance through the opsin GPCR pathway or by regulating the transcriptional levels of CYP6A1, which finally leads to resistance, still needs in-depth research.

In this study, the arrestin gene was expressed at every developmental stage in *Cx. pipiens pallens* and varied in different developmental stages. This observation suggests that arrestin is important to cell growth and development. The expression levels of arrestin gene were significantly different between DM-resistant and DM-susceptible strains at egg, pupae, male, and female stages, but not in larval stages, implying that arrestin possibly plays a role in DM resistance at all stages except larval stages. Interestingly, the expression level of arrestin in males was higher than that in female adults of DS strain. This result coincides with the results obtained from *Anopheles gambiae*[53]. Therefore, arrestin displays preferential function involved in male-specific activities. Moreover, arrestin, as a multifunctional protein, performs different functions at different stages. However, the specific mechanisms need to be further elucidated.

## Conclusions

In this study, we have cloned the complete arrestin gene from *Cx. pipiens pallens* and found the transcriptional level of arrestin was up-regulated at the egg, pupa, and adult stages in DR strain than in DS strain. Our study provides the first evidence that arrestin might be associated with insecticide resistance in *Cx. pipiens pallens*. In addition, whether arrestin participates in resistance through the opsin GPCR pathway or by regulating the transcriptional levels of CYP6A1, which finally leads to resistance, still needs further study.

## Abbreviations

DM, Deltamethrin; DR, Deltamethrin-resistant; DS, Deltamethrin-susceptible; DDT, Dichlorodiphenyltrichloroethane; CYP6A1, Cytochrome P450 6A1.

## Competing interests

The authors declare that they have no competing interests related to this article.

## Authors’ contributions

CLZ and YS conceived and designed the research. YS and ZP performed the experiments and analyzed the data. XYY, CC, JY, LNS, SCH, DZ, XLC, WJW, BS, DHZ and LM contributed materials and helped in study implementation. YS and ZP wrote and revised the manuscript. All authors read and approved the final version of the manuscript.

## Supplementary Material

Additional file 1 **Figure S1.** Real-time PCR analysis of mRNA level of arrestin in DR strain and DS strain of *Cx. pipiens pallens*. The relative expression of arrestin in DS strain was considered as background level or 1, and the mRNA expression of arrestin is shown as the relative value against *β*-actin. Results are expressed as mean ± standard error (SE) of three independent experiments. *p < 0.05 compared with DS. (TIFF 228 kb) Click here for file

Additional file 2 **Figure S2.** The nucleotide and deduced amino acid sequences of arrestin from *Cx. pipiens pallens*. The deduced amino acid sequence is presented below the nucleotide sequence in single-letter code. The poly (A) in the 3’-untranslated region are in bold letters. The initial code “ATG”, the termination codon “TAG,” and the tailing signal sequence “AATAAA” in the 3’-untranslated region are in bold letters. GenBank ID: HQ833831. (TIFF 453 kb) Click here for file

Additional file 3 **Figure S3.** Amino acid sequence alignment of arrestin gene from *Cx. pipiens pallens* and other arrestin species. Asterisks indicate identical amino acid and dots indicate similar amino acids. Abbreviations and GenBank accession no.: *Cx. pipiens pallens*, HQ833831.2; *Cx. quinquefasciatus*, XM_001844435.1; *Ae. aegypti*, XM_ 001663682.1; *Anopheles gambiae*, AY017417.1; *D. simulans*, XM_002079727; *D. yakuba*,XM_002090394.1; *H. sapiens*, NM_004313.3; *M. musculus*, NM_145429.4; *Danio rerio*, NM_001159822.1. (TIFF 3205 kb) Click here for file

Additional file 4 **Figure S4.** Phylogenetic relationships of arrestin between *Cx. pipiens pallens* and other species. Abbreviations and GenBank Accession No. are the same as that of Additional file 2: Figure S2. Click here for file
