# Supplementary figures and images for: Functional characterization of an arrestin gene on insecticide resistance of Culex pipiens pallens
Source: Parasit Vectors. 2012 Jul 6;5:134. doi: 10.1186/1756-3305-5-134 (PMC3425237; doi:10.1186/1756-3305-5-134)

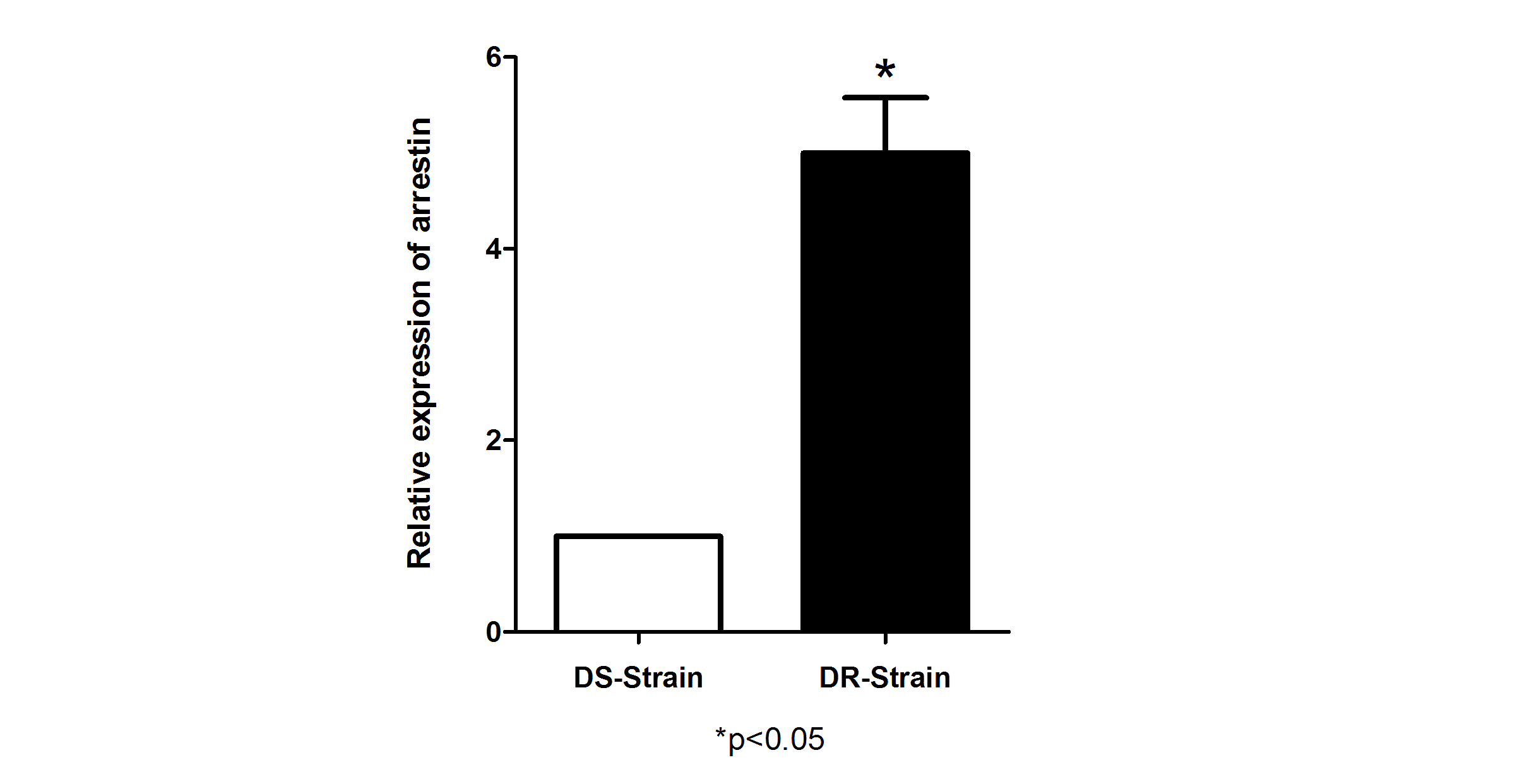

Supplement: Additional file 1 — Figure S1. Real-time PCR analysis of mRNA level of arrestin in DR strain and DS strain of Cx. pipiens pallens. The relative expression of arrestin in DS strain was considered as background level or 1, and the mRNA expression of arrestin is shown as the relative value against β-actin. Results are expressed as mean ± standard error (SE) of three independent experiments. *p < 0.05 compared with DS. (TIFF 228 kb) [file 1756-3305-5-134-S1.tiff]

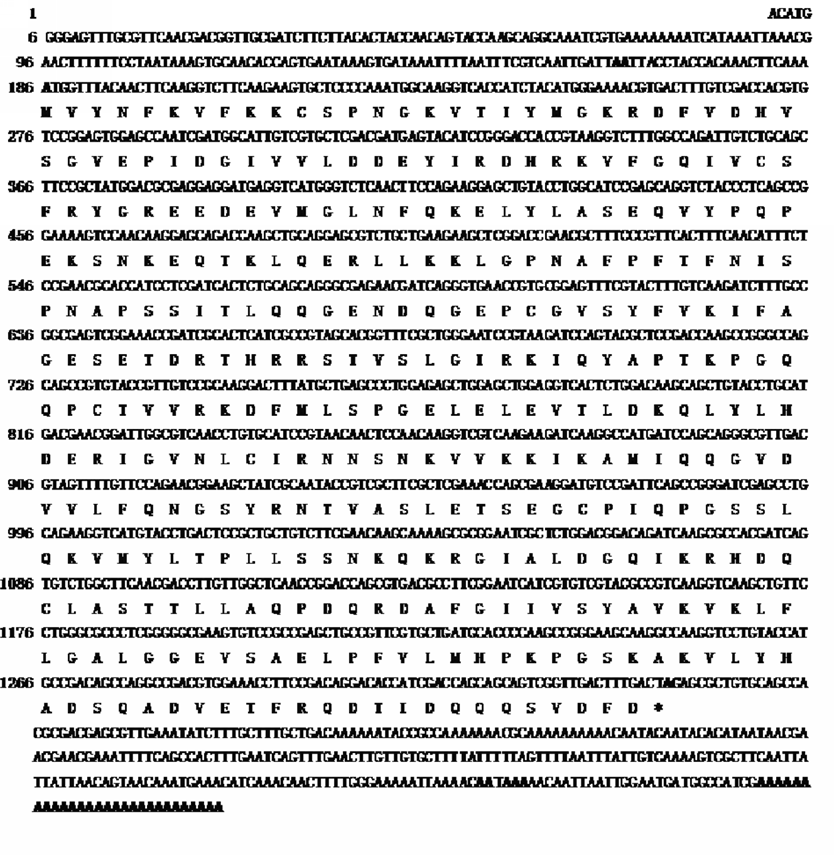

Supplement: Additional file 2 — Figure S2. The nucleotide and deduced amino acid sequences of arrestin from Cx. pipiens pallens. The deduced amino acid sequence is presented below the nucleotide sequence in single-letter code. The poly (A) in the 3’-untranslated region are in bold letters. The initial code “ATG”, the termination codon “TAG,” and the tailing signal sequence “AATAAA” in the 3’-untranslated region are in bold letters. GenBank ID: HQ833831. (TIFF 453 kb) [file 1756-3305-5-134-S2.tiff]

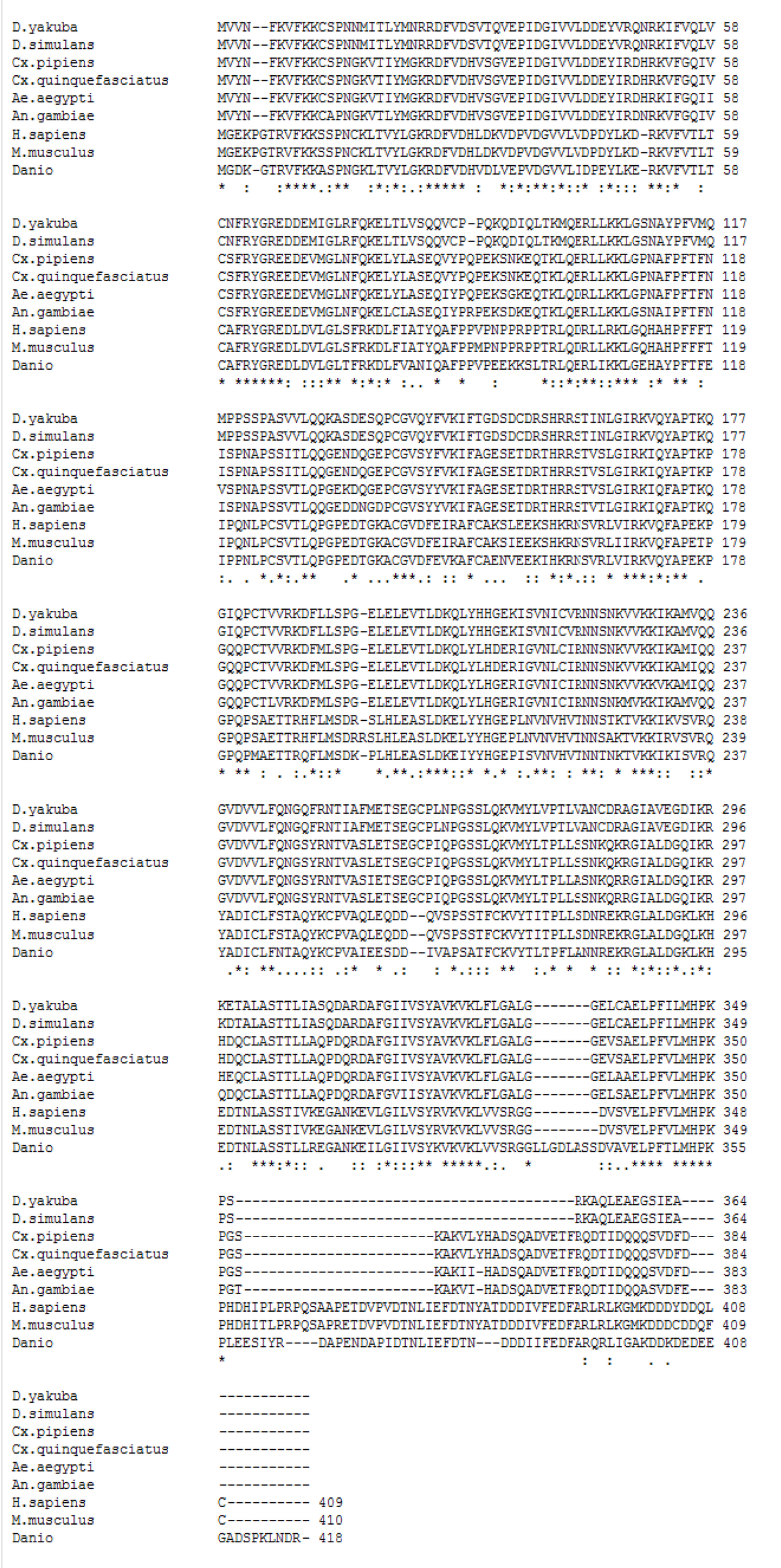

Supplement: Additional file 3 — Figure S3. Amino acid sequence alignment of arrestin gene from Cx. pipiens pallens and other arrestin species. Asterisks indicate identical amino acid and dots indicate similar amino acids. Abbreviations and GenBank accession no.: Cx. pipiens pallens, HQ833831.2; Cx. quinquefasciatus, XM_001844435.1; Ae. aegypti, XM_ 001663682.1; Anopheles gambiae, AY017417.1; D. simulans, XM_002079727; D. yakuba,XM_002090394.1; H. sapiens, NM_004313.3; M. musculus, NM_145429.4; Danio rerio, NM_001159822.1. (TIFF 3205 kb) [file 1756-3305-5-134-S3.tiff]

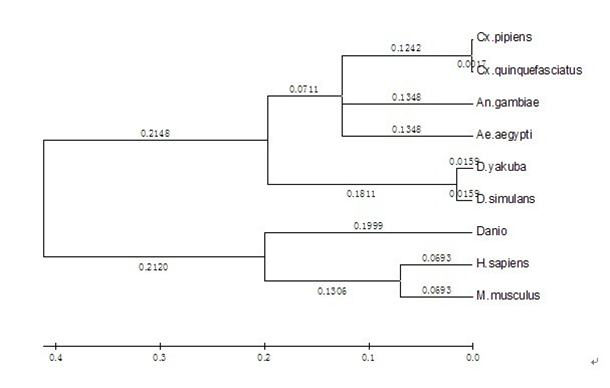

Supplement: Additional file 4 — Figure S4. Phylogenetic relationships of arrestin between Cx. pipiens pallens and other species. Abbreviations and GenBank Accession No. are the same as that of Additional file 2: Figure S2. [file 1756-3305-5-134-S4.tiff]
